# Inhaled corticosteroids use in childhood respiratory diseases: an italian survey on pediatricians’ prescription habits

**DOI:** 10.1186/s13052-021-00988-8

**Published:** 2021-02-15

**Authors:** Giovanni Cerimoniale, Paolo Becherucci, Maria Carmen Verga, Giuseppe Di Mauro, Luciana Indinnimeo, Alberto Villani, Mariangela Tosca, Gian Luigi Marseglia, Marzia Duse, Paolo Biasci, Mattia Doria, Diego Peroni, Giorgio Piacentini, Maria Di Cicco, Gabriella Pozzobon, Riccardo Lubrano

**Affiliations:** 1 Pediatric Primary Care, National Pediatric Health Care System, Rome, Italy; 2 SICuPP (Società Italiana delle Cure Primarie Pediatriche), Milan, Italy; 3 SIPPS (Società Italiana di Pediatria Preventiva e Sociale), Milan, Italy; 4 Pediatric Department, “Sapienza” University, Rome, Italy; 5 SIP (Società Italiana di Pediatria), Rome, Italy; 6 Infectious Disease Unit, Academic Pediatric Department, Pediatric Hospital “Bambino Gesù”, Rome, Italy; 7 Department of Pediatrics, Pulmonology and Allergy Units, “Giannina Gaslini” Institute, Genoa, Italy; 8 SIAIP (Società Italiana di Allergologia ed Immunologia Pediatrica), Milan, Italy; 9 Pediatrics Clinic, Pediatrics Department, Policlinico San Matteo, University of Pavia, Pavia, Italy; 10 FIMP (Federazione Italiana Medici Pediatri), Rome, Italy; 11 Department of Clinical and Experimental Medicine, Section of Pediatrics, University of Pisa, Pisa, Italy; 12 Department of Surgical Sciences, Dentistry, Gynecology and Pediatrics, Pediatric Division, University of Verona, Verona, Italy; 13 SIMRI (Società Italiana per le Malattie Respiratorie Infantili), Naples, Italy; 14 Department of Pediatric, IRCCS San Raffaele Hospital, Milan, Italy; 15 SIMA (Società Italiana di Medicina dell’Adolescenza), Palermo, Italy; 16 Pediatrics and Neonatology Unit, “Sapienza” University, Latina, Italy; 17 SIMEUP (Società Italiana di Medicina Emergenza Urgenza Pediatrica), Milan, Italy

**Keywords:** Inhaled corticosteroids, Allergic rhinitis, Asthma, Preschool wheezing, Laryngitis, Children

## Abstract

**Background:**

A national consensus document on inhaled corticosteroids (ICS) use in childhood, produced by the main Italian pediatric scientific societies, has been recently released. The aim of this study was to gather information on the current pediatricians’ ICS prescription habits in Italy for the management of the most common pediatric respiratory diseases, namely allergic rhinitis (AR), asthma, preschool wheezing and laryngitis.

**Methods:**

From the 1st October 2018 to the 31st January 2019 a link to an online questionnaire was sent monthly through a newsletter to the members of the Italian Society of Pediatrics. The questionnaire included 18 items on ICS use in the most common pediatric respiratory diseases. Data collection and reporting was based on STROBE Statement Checklist for cross-sectional studies.

**Results:**

One thousand-two questionnaires were returned from primary care pediatricians (39.1%), hospital pediatricians (38.7%), private practicers (16.4%), university pediatricians (3.1%) and Pediatrics residents (2.7%). We found a good adherence to the international guidelines on AR, with prevalent use of oral antihistamine (60.6%) in the secretive phenotype and nasal ICS in the obstructive phenotype (64.8%). In asthma exacerbations ICS are not used in 53.4% of cases, but they are used at high dose in 27.9% and at low dose in 18.7% of cases. In intermittent asthma, ICS are not chosen as a daily controller therapy in 54.1% of cases, while they are chosen as a low dose daily therapy in 44.5% of cases (high dose in 1.4%). In children with persistent asthma, ICS are chosen as a daily low dose therapy in 67.4% of cases and as a daily high dose therapy in 31%. In the management of preschool wheezing, when a long-term treatment is needed, ICS are chosen both alone and in association with antileukotrienes in 71.4% of cases. Children affected by recurrent asthma exacerbations and wheezing are closely followed up, in particular by their primary care pediatricians. The preference for certain molecules in the treatment of different respiratory diseases also emerged.

**Conclusions:**

Pediatricians’ ICS prescription habits in Italy should be improved, especially in the management of asthma. Future surveys on a more numerous sample will be useful to analyze differences in prescription habits on the basis of pediatricians’ work settings and geographical distribution.

**Supplementary Information:**

The online version contains supplementary material available at 10.1186/s13052-021-00988-8.

## Background

Respiratory allergic diseases represent a significant health problem in childhood. Epidemiological data show that in Italy the prevalence of allergic rhinitis (AR) varies from 5 to 15% in children aged 6–14 years, while the prevalence of asthma varies from 9.3% in children to 10.3% in adolescents. Severe asthma is reported in 1.6% of children and 12.3% of adolescents [1]. Asthma and AR have a huge impact on health care systems in terms of costs: as far as AR, it has been calculated that in Europe between 55 and 151 million euros are lost each year, including parents’ absence from work and reduced productivity [2]. In Italy, direct costs of bronchial asthma, which has been included in the Italian list of chronic and invalidating diseases in 1999 [3], are estimated to be between 1 and 2% of overall health care system costs. The frequency of hospitalization is also remarkable, as recently confirmed by the OSMED report [4]. Therefore, it is not surprising that the most prescribed categories of drugs in children are those related to the treatment of the airways (35.6%): as a matter of fact, in the list of the first 20 most prescribed drugs in Italy there are five inhaled corticosteroids (ICS) [4]. ICS are widely used in respiratory diseases treatment also in adults because they are highly effective and allow to choose whether to treat upper and/or lower airways on the basis of the dimensions and rheological properties of the particles aerosolized by different devices using different molecules. Moreover, administration of corticosteroids by inhalation avoids the use of the systemic route, with consequent advantages in terms of safety [5]. However, the fact that ICS are commonly prescribed in our country implies cost issues and potential health risks for children related to recurrent and / or prolonged corticosteroids exposition [6–9]. Consequently, in 2019 the representatives of the main Italian pediatric scientific societies and professional associations (Additional file 1) released a national consensus document to provide a valid reference paper to promote the appropriate use of ICS [10]. Moreover, in order to obtain an updated and reliable picture on the current ICS prescription habits in Italy, they carried out a web-based survey involving pediatricians from every part of the country. Here, we report the main results from this survey.

## Methods

### Study design and settings

A cross-sectional and observational study was conducted from the 1st October 2018 to the 31st January 2019 sending monthly by email a link to an online questionnaire to all the pediatricians who were active members of the Italian Society of Pediatrics (SIP). The study was conducted following the STROBE Statement Checklist for cross-sectional studies (Additional file 2).

### Sample size and sampling

The Italian health care system provides pediatric primary care assistance for each child since birth and till 14 years of age for free. Even if parents can choose whether to switch the assistance to a general practitioner starting from 6 years of age, almost all of them prefer to continue the pediatric assistance as long as possible. In special cases, such as chronic and/or rare diseases, pediatric assistance can be prolonged up to 16 years. When hospitalization is needed, almost every child is admitted in Pediatrics departments up to the age of 14–18 years, depending on the Region. Therefore, the vast majority of children are evaluated and treated by pediatricians in our country. SIP allows Italian pediatricians, independently on their work settings or status, to become members on a voluntary basis and following the payment of a yearly fee. In 2018 there were about 17.700 pediatricians in Italy and SIP had almost 9000 members, including primary care, private practice, hospital and university pediatricians. The latter have a dual role as hospital pediatricians (working in University hospitals) and university professors and/or researchers. Pediatrics residents can become SIP members during their training years, too. We estimated a sample size of at least 563 participants for a confidence level of 95% and a precision of 4% to be representative of SIP members.

### Measures and study variables

A not yet validated and easy-to-fill questionnaire was created and approved by a working group consisting of the representatives from all the Italian scientific societies and associations involved in the national consensus document on ICS use in childhood (Additional file 1) [10]. The questionnaire was anonymous and only age, sex, geographical area of activity and work setting were recorded. The main respiratory diseases included in the questionnaire were laryngitis, AR, asthma and preschool wheezing and only the most commonly prescribed drugs and molecules were cited. To avoid double or multiple compilations a control system was included, so that only a single access was allowed. The survey consisted of 18 multiple-choice questions (Table 1) and answering to all of the questions was mandatory in order to complete the survey. Detailed instructions to correctly fill the form were provided. The frequency of ICS prescription (items 7, 11 and 17) was defined as follows: “very frequently” = in more than 70% of the patients; “frequently” = between 40 and 70% of the patients; “sometimes” = in less than 40% of the patients. Regarding the influence of the parents’ opinion (item 18), it was defined as follows: “very much” = it made the pediatrician change his/her mind; “much” = the opinion was taken into account when choosing drugs and devices; “a little” = it didn’t influence the pediatrician’s decision. Regarding the definition of “high” and “low dose” ICS in asthma treatment, the tables from GINA recommendations were provided for children < 5 years, between 6 and 11 years and > 12 years [11], specifying that, in order to make filling the form easier, both “high” and “medium” dose should have been considered as “high dose”.

**Table 1:** Items included in the “Use of ICS in childhood” questionnaire

Use of ICS in childhood									
**1. Your age**	**2. Sex**	**3.Your activity**			**4. Geographical area where you work**	**5. In allergic rhinitis with prevalent secretory component (rhinorrhea, sneezing) which drug do you use first?**			**6. In allergic rhinitis with prevalent obstructive component (nasal obstruction, not sneezing) which drug you use first?**
a) 30–34 yrs b) 35–39 yrs. c) 40–44 yrs d) 45–49 yrs e) 50–54 yrs f) 55–59 yrs g) ≥ 60 yrs	a) Male b) Female	a) Primary care pediatrician b) Hospital pediatrician c) University pediatrician d) Private practice pediatrician e) Pediatrics resident			a) Abruzzo b) Basilicata c) Calabria d) Campania e) Emilia Romagna f) Friuli Venezia Giulia g) Lazio h) Liguria i) Lombardy j) Marche k) Molise l) Piedmont m) Apulia n) Sardinia o) Sicily p) Tuscany q) Trentino Alto Adige r) Umbria s) Aosta Valley t) Veneto	a) Nasal ICS b) Oral antihistamine c) Nasal ICS + oral antihistamine d) None of the above			a) Nasal ICS b) Oral antihistamine c) Nasal ICS + oral antihistamine d) None of the above
**7. When prescribing an ICS to treat allergic rhinitis, how frequently do you choose the following molecules?**					**8. Do you use ICS to treat asthma exacerbations?**	**9. Do you prescribe ICS as a maintenance therapy in asthmatics with less than 2 exacerbations / week and with night symptoms less than twice a month?**			**10. Do you prescribe ICS as a maintenance therapy in asthmatics with more than 2 exacerbations / week and with night symptoms more than twice a month?**
	**Very frequently**	**Frequently**	**Sometimes**	**Never**	a) No b) Yes, low dose c) Yes, high dose	a) No b) Yes, at low dose c) Yes, at high dose			a) No b) Yes, at low dose c) Yes, at high dose
Beclomethasone									
Budesonide									
Flunisolide									
Fluticasone									
Mometasone									
**11. When prescribing an ICS as a maintenance therapy for asthma, how frequently do you choose the following molecules?**					**12. When a maintenance treatment is indicated in asthma, when do you plan to re-evaluate the patient after starting ICS administration?**	**13. To manage recurrent wheezing episodes in preschoolers, which therapeutic strategy do you choose?**			**14. When you believe a maintenance treatment is needed in preschool recurrent wheezing, which drug do you choose?**
	**Very frequently**	**Frequently**	**Sometimes**	**Never**	a) One month b) Two months c) Three months	a) I promptly treat every single acute episode b) I treat the acute episode and prescribe a maintenance therapy			a) ICS b) Antileukotrienes c) ICS and antileukotrienes d) Other drugs
Beclomethasone									
Budesonide									
Flunisolide									
Fluticasone									
Mometasone									
**15. When a maintenance treatment is indicated in preschool recurrent wheezing, when do you plan to re-evaluate the patient after starting drugs administration?**	**16. Which device do you suggest to administer ICS?**			**17. When prescribing an ICS to treat laryngitis, how frequently do you choose the following molecules?**					**18. When you decide to start a long term treatment for wheezing, asthma or allergic rhinitis, how much the parents’ opinion influence your decisions (molecules, devices, timing, etc).**
a) One month b) Two months c) Three months d) After the winter period	a) Nebulizer b) pMDI + spacer c) I choose case by case, on the basis of the family and child degree of collaboration d) Nebulizer in preschoolers and pMDI + spacer in older children e) Dry powder inhalers				**Very frequently**	**Frequently**	**Sometimes**	**Never**	a) Very much b) Much c) A little d) At all
				Beclomethasone					
				Budesonide					
				Flunisolide					
				Fluticasone					
				Mometasone					

### Statistical analysis

We performed a descriptive analysis of the collected data to evaluate pediatricians’ ICS prescription habits as a whole. Analysis of subgroups based on age, geographical area or work setting of the participants was beyond the scope of our work. Data were expressed as numbers and percentages.

## Results

### Study population

At the end of the survey 1002 questionnaires were returned; 650 participants were female (64.9%). The age distribution of the participants is reported in Table 2. Most of the questionnaires were from primary care pediatricians (39.1%) and hospital pediatricians (38.7%), while 16.4% were from private practicers, and only 3.1 and 2.7% were respectively from university pediatricians and Pediatrics residents (Table 2). Participants were distributed throughout the national territory and each Region was represented (Table 3). No data was missing since answering all the questions was mandatory in order to complete the survey.

**Table 2:** – Demographic characteristics of the survey responders (*n* = 1002)

	*n* (%)
**Sex**	
*Female*	650 (64.9)
*Male*	352 (35.1)
**Age distribution**	
30–34 years	108 (10.8)
35–39 years	133 (13.3)
40–44 years	109 (10.9)
45–49 years	64 (6.4)
50–54 years	113 (11.3)
55–59 years	170 (16.9)
≥ 60 years	305 (30.4)
**Work setting**	
Primary care pediatricians	392 (39.1)
Hospital pediatricians	388 (38.7)
Private practice pediatricians	164 (16.4)
University pediatricians	31 (3.1)
Pediatrics residents	27 (2.7)

**Table 3:** - Geographical origin of the participants (*n* = 1002)

Italian Region	Participants (***n***)
Abruzzo	17
Basilicata	6
Calabria	10
Campania	102
Emilia Romagna	81
Friuli Venezia Giulia	24
Lazio	124
Liguria	23
Lombardy	184
Marche	29
Molise	6
Piedmont	60
Apulia	82
Sardinia	24
Sicily	62
Tuscany	60
Trentino Alto Adige	14
Umbria	17
Aosta Valley	4
Veneto	73

### Allergic rhinitis

**Fig. 1:**
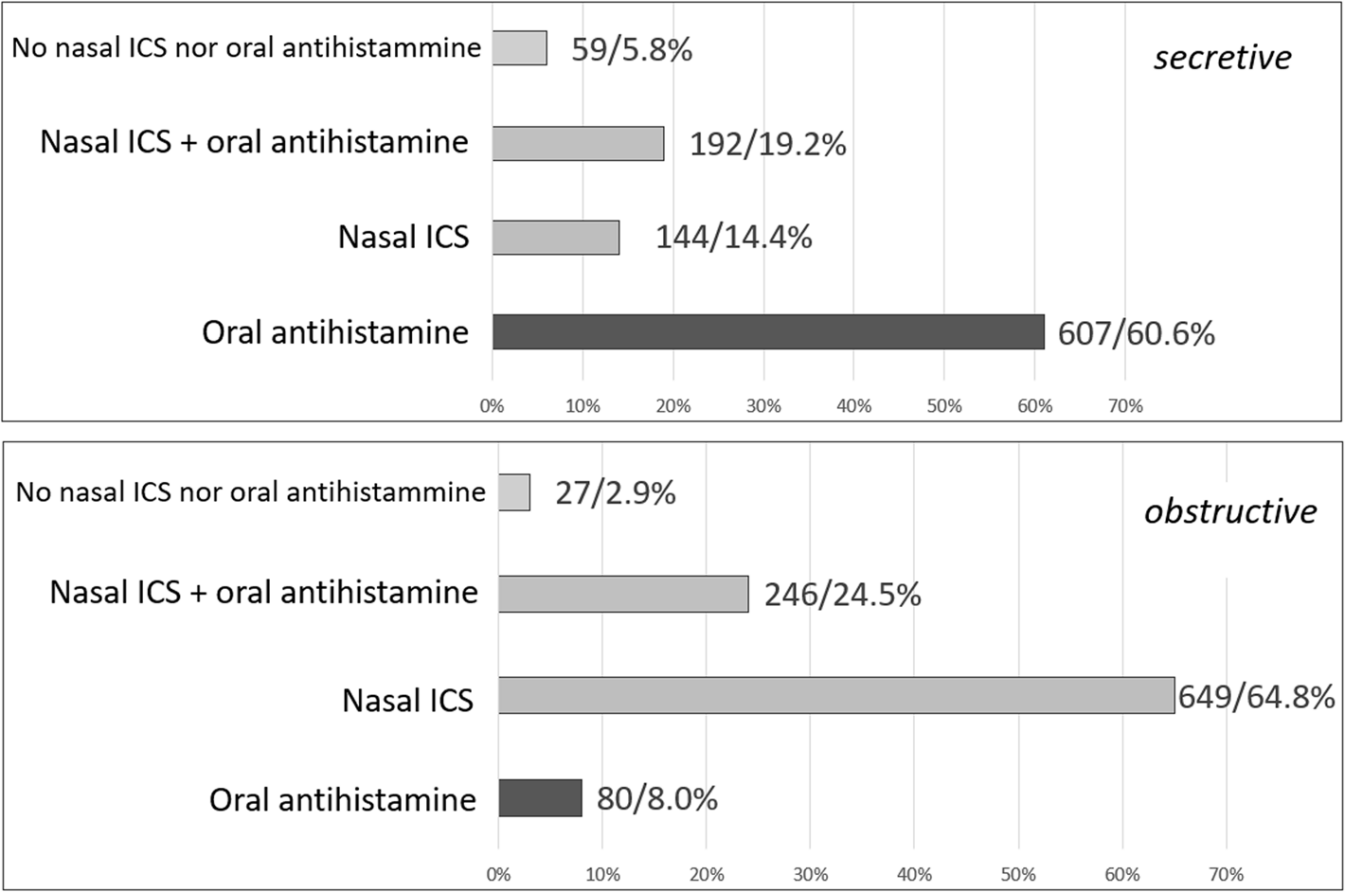
Treatments used in AR with prevalent secretive or obstructive component

**Fig. 2:**
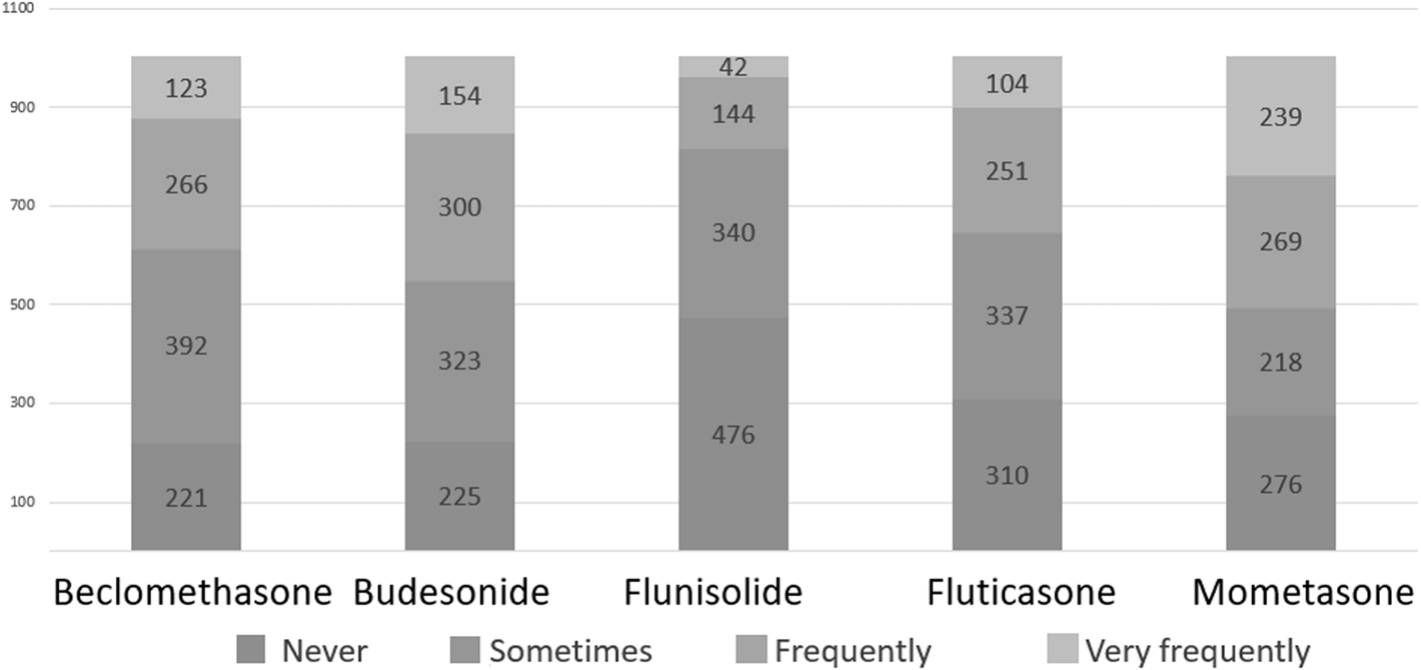
ICS use in the treatment of AR

To the question “*In allergic rhinitis with prevalent secretory component (rhinorrhea, sneezing) which drug do you use first?*”, pediatricians showed a clear preference for oral antihistamine (*n* = 607, 60.6%) rather than nasal ICS (*n* = 144, 14.4%), while the association between antihistamine and nasal ICS was chosen by 192 responders (19.2%) (Fig. 1). To the question “*In allergic rhinitis with prevalent obstructive component (nasal obstruction, not sneezing) which drug you use first?*”, 64.8% (*n* = 649) of the participants chose nasal ICS, while only 8% (*n* = 80) chose oral antihistamine. The combination therapy was chosen by 246 responders (24.5%) (Fig. 1). As far as the molecules, the most prescribed (frequently + very frequently) nasal steroid was mometasone (50.7%), followed by budesonide (45.3%), beclomethasone (38.8%) and fluticasone (35.4%) (Fig. 2).

### Asthma

**Fig. 3:**
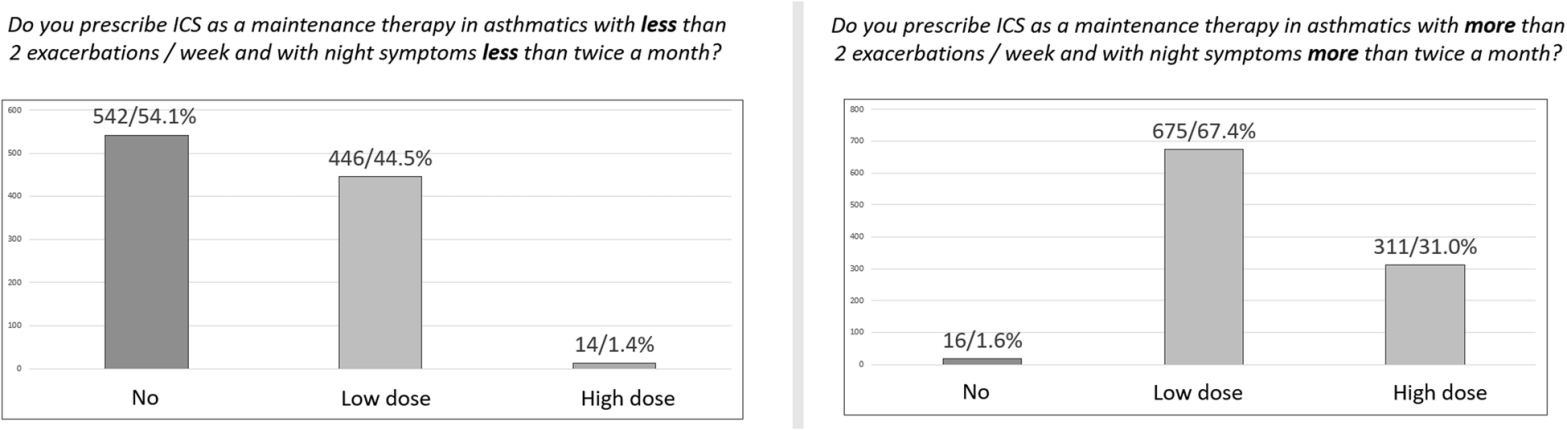
ICS use in the treatment of asthma on the basis of the frequency of exacerbations

**Fig. 4:**
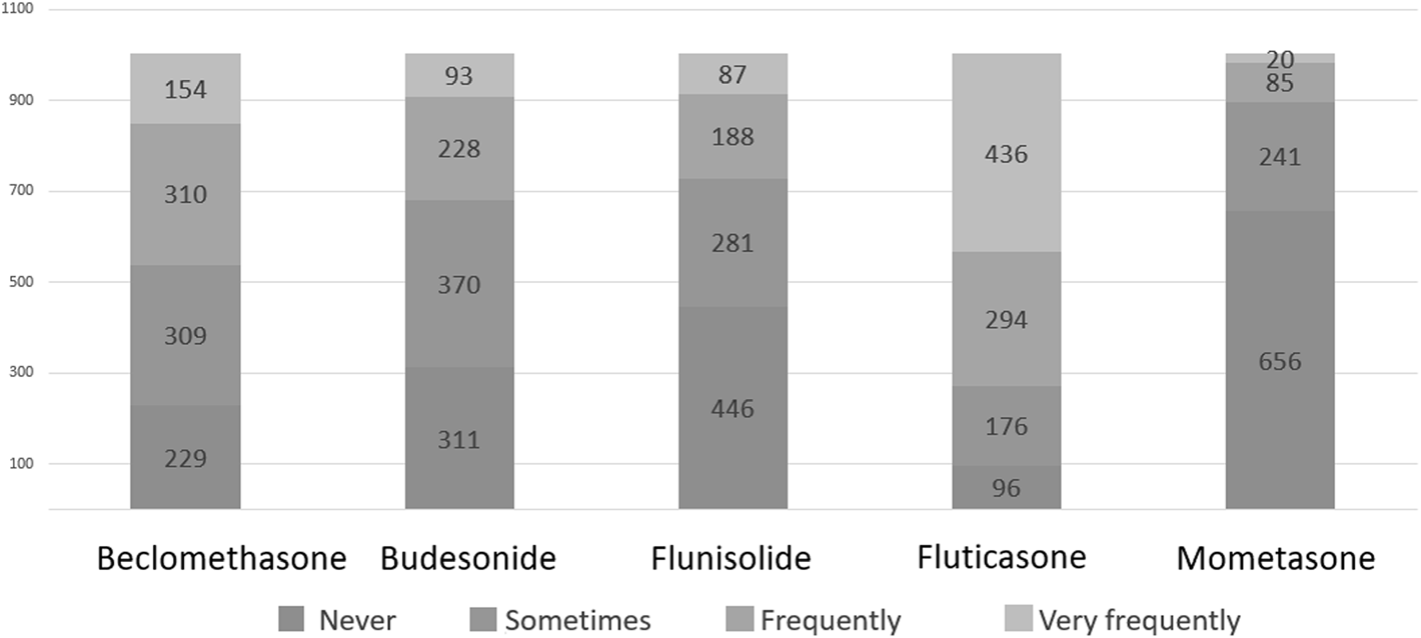
ICS use in asthma maintenance treatment

To the question “*Do you use ICS to treat asthma exacerbations?*”, 53.4% of the participants denied, but 27.9% declared to use ICS at high dose and 18.7% at low dose. Two questions were aimed at investigating how recurrent asthma is managed on the basis of the frequency of exacerbations, distinguishing recurrence in two main categories: 1) *infrequent asthma* (acute episodes less than twice a week and night-time symptoms less than twice a month); 2) *frequent (persistent) asthma* (acute episodes more than twice a week and nocturnal symptoms more than twice a month) [12]. In the first case, 54.1% of the participants declared that they did not use ICS, 44.5% that they used ICS at low dose and 1.4% that they used ICS at high dose. In persistent asthma, almost all the participants declared to use ICS: 67.4% of the participants chose ICS at low dose while 31.0% chose ICS at high dose (Fig. 3). The most frequently prescribed ICS (frequently + very frequently) as a maintenance therapy was fluticasone (72.8%), followed by beclomethasone (46.3%), budesonide (32.0%), flunisolide (27.4%) and mometasone (10.5%) (Fig. 4). To the question “*When a maintenance treatment is indicated in asthma, when do you plan to re-evaluate the patient after starting ICS administration?”* 48% of the participants answered after one month (*n* = 481), 23% after two months (*n* = 231), 29% after 3 m (*n* = 290).

### Preschool wheezing

**Fig. 5:**
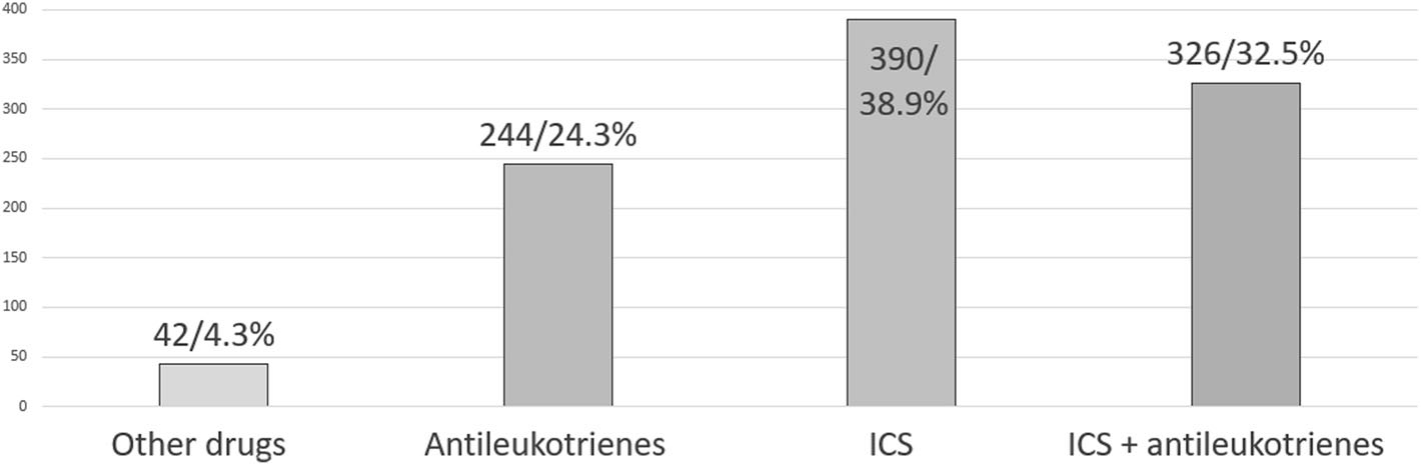
Strategies to manage preschool wheezing

To the question: “*To manage recurrent wheezing episodes in preschoolers, which therapeutic strategy do you choose?”*, 48.8% of the participants replied that they just used to manage the acute episode (489) while 51.2% chose a controller therapy (513). To the question: “*When you believe a maintenance treatment is needed in preschool recurrent wheezing, which drug do you choose?*”, we found that ICS were the most used drugs (38.9%), followed by the ICS + antileukotrienes association (32.5) and antileukotrienes alone (24.3%) (Fig. 5). Finally, to the question: “*When a maintenance treatment is indicated in preschool recurrent wheezing, when do you plan to re-evaluate the patient after starting drugs administration?”*, 39% of the participants declared that they used to re-evaluate the child within 1 month, 20.8% within 2 months, 25.6% within 3 months, while 14.6% chose to prescribe a long-term treatment, starting in autumn and ending at the end of winter.

### Other questions

**Fig. 6:**
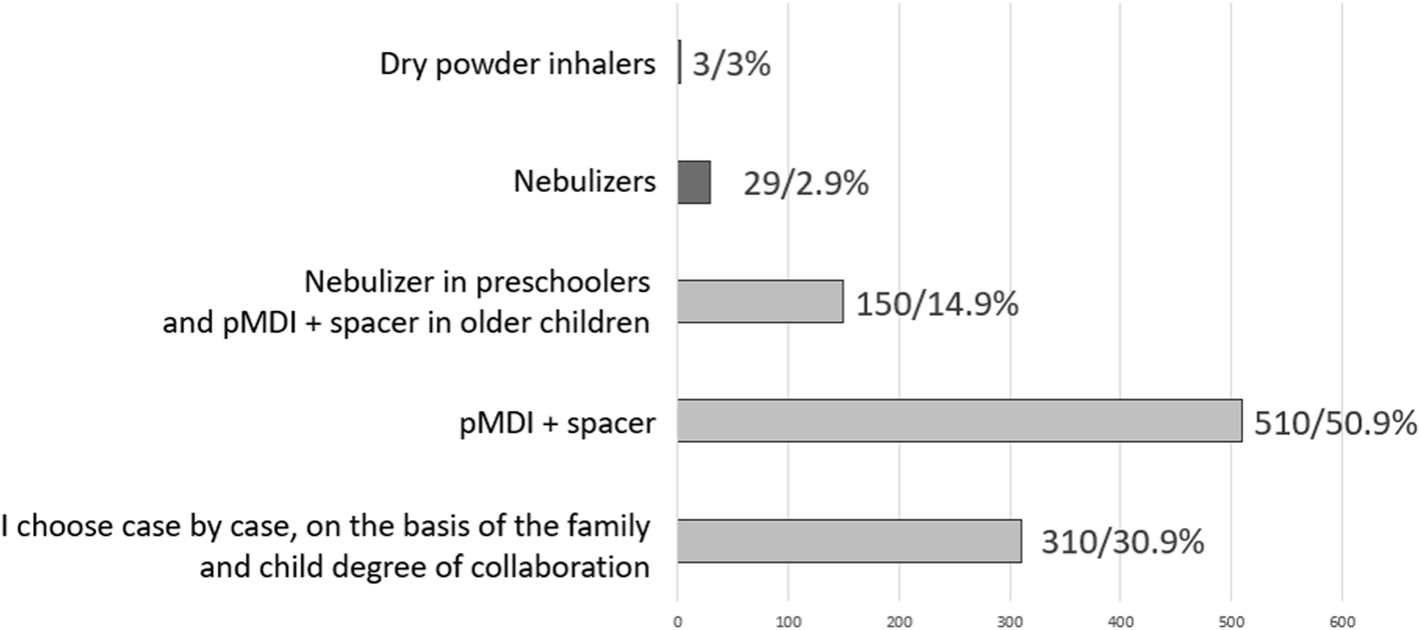
Devices most commonly prescribed to administer ICS for lower airways treatment

**Fig. 7:**
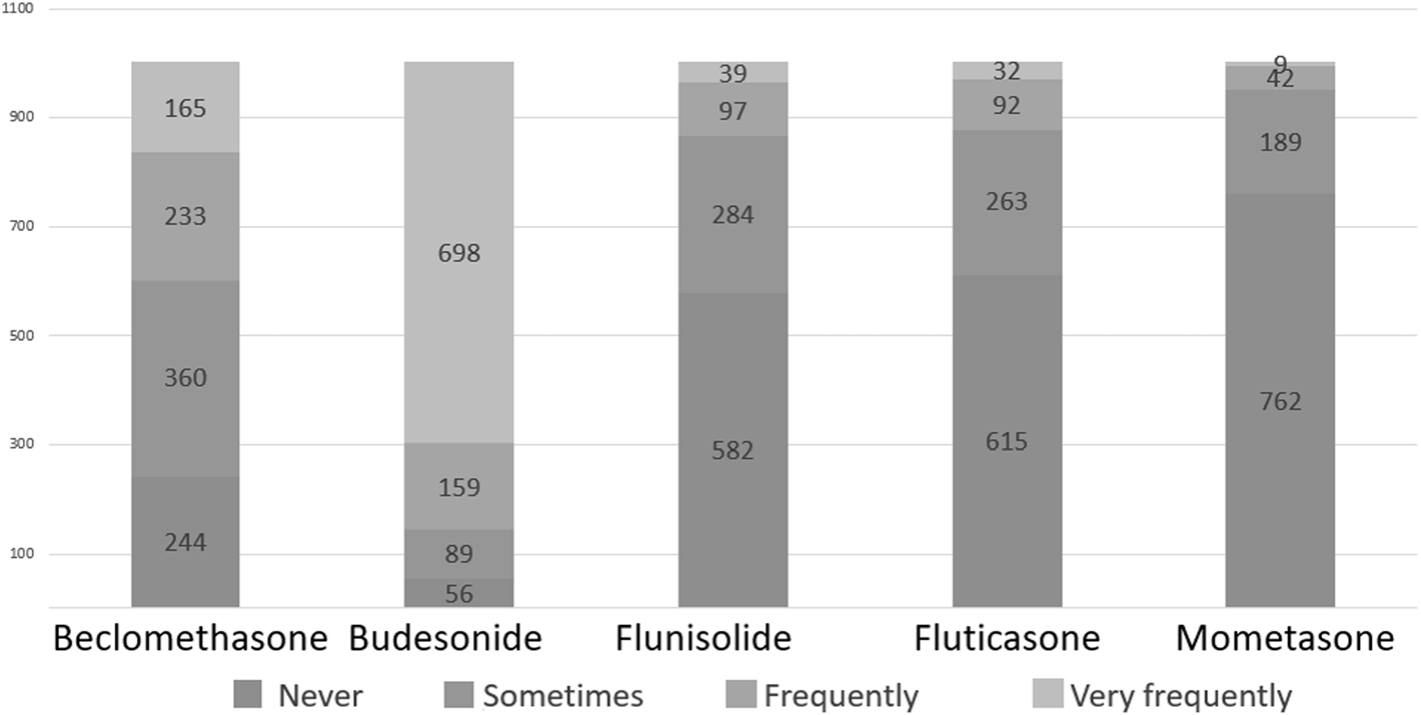
ICS use in the treatment of laryngitis

To the question: “*Which device do you suggest to administer ICS?*”, 50.9% of the pediatricians declared to prescribe a pMDI + spacer, while 30.9% chose the device on the basis of the preferences and degree of collaboration of the child and the family; 14.9% of the participants used to recommend nebulizers in preschoolers and pMDI + spacer in older children and only 3 participants declared to be used to recommend dry powder inhalers (Fig. 6). As far as the treatment of acute laryngitis is concerned, the most used ICS (very frequently + frequently) was budesonide (85.5%), followed by beclomethasone (39.7%), flunisolide (13.6%), fluticasone (12.4%) and mometasone (5.1%) (Fig. 7). To the question: “*When you decide to start a long-term treatment for wheezing, asthma or allergic rhinitis, how much the parents’ opinion influence your decisions (molecules, devices, timing, etc)”* 45.2% of the participants answered “a little”, 38.3% “much”, 10.6% “not at all”, 5.9% “very much”.

## Discussion

To our knowledge, our study is the first having gathered information on pediatricians’ ICS prescription habits in Italy for the management of the most common pediatric respiratory diseases. The pediatricians who participated to the survey left very good feedbacks, supporting the importance of the topic for pediatricians and the usefulness of surveys as tools to investigate clinical practices. We collected data from almost 10% of SIP members: as expected, most of the questionnaires were filled out by primary care and hospital pediatricians who are usually keener to analyze clinical practice issues rather than research issues. We found that in our country the treatment of AR is in agreement with international guidelines, with a greater use of antihistamine in AR with prevalent secretive component, and of nasal ICS in AR with prevalent obstructive component [2]. We chose this definition of AR phenotypes not including the recent classification of intermittent and persistent AR and non-allergic rhinitis to reduce the difficulty and time required to fill the questionnaire. As far as asthma is concerned, the aims of the survey were to evaluate the approach to a child with asthma exacerbation as well as the management of patients with persistent asthma. We found that even if 53.4% of the pediatricians avoid the use of ICS in asthma exacerbations, the remaining 46.6% prefer to use ICS both at high dose and at low dose in spite of the fact the main national and international guidelines agree in recommending not to use ICS to treat asthma exacerbations [11–15]. Nevertheless, some good methodological quality systematic reviews and meta-analysis have shown a possible role of ICS in the treatment of exacerbations [16], but these works have been carried out in the setting of emergency departments, using very high dose of ICS mostly administered by nebulizers, with conflicting results [17, 18]. As far as maintenance therapy, more than 67% of the participants used to prescribe low dose ICS in persistent asthma, in agreement with the guidelines. Conversely, 31% of the participants used to prescribe high dose ICS in these patients. Less than half of the responders started a maintenance treatment in intermittent asthma. However, high doses were suggested also in these cases, demonstrating that pediatricians show an excessive concern on the influence of the single episodes on the functional integrity of the airways and on the quality of life of the children. It is not possible to understand whether all these treatments were recommended in the contest of a step-down or step- up strategy, since the assessment of the behavior of pediatricians in the management of the long-term therapies was beyond the aims of our study. Notably, even if most of the guidelines suggest to reassess the patients after 3 months of therapy, more than 45% of the responders re-evaluated their patients after 1 month of maintenance treatment: this data may be influenced by the high response rate of primary care pediatricians, since they are particularly keen to follow up their children more closely. As for preschool wheezing, the optimal therapeutic strategy is far from been identified: a maintenance therapy should be recommended in children with recurrent episodes and/or risk factors for recurrence such as family and personal history of allergy/atopy, in order to reduce the number of hospitalizations and the burden for the children and their family. Early schooling, the presence of associated diseases, tobacco exposure, siblings going to school and overcrowding at home should be evaluated too. However, when long-term treatment is needed, pediatricians should always look for the lowest effective dose to avoid an excessive use of drugs [19, 20]. We must admit that a definition of wheezing and its recurrence as well as the related risk factors should have been included in the survey in order to obtain more realistic answers. We didn’t find a clear preference for a single drug in the prevention of wheezing recurrence, confirming a well-known difficulty in phenotyping wheezing [21–24]. Nevertheless, ICS were the most prescribed drugs in these patients (38.9%), but also antileukotrienes were commonly used both alone (24.3%) or in association with ICS (32.5%). As far as the choice of a device to treat the lower airways, only 50.9% of the pediatricians follows the guidelines choosing a pMDI + spacer. However, 30.9% of the pediatricians declared to choose the device on the basis of the family and patient’s preferences. Regarding this latter point, responses on the item related to the influence of the parents’ opinion are not easy to evaluate, since most of the participants answered that they were “a little” or “much” influenced by them: whether these answers are related to real needs of the family or the doctor’s need to obtain adherence, is not known [7, 25–28]. Our study results show that Italian pediatricians use to prescribe some corticosteroid molecules more than others to treat different diseases: as far as budesonide for the treatment of laryngitis, this strategy is supported by its well-known vasoconstrictive effect [29, 30]. As for asthma, AR and preschool wheezing, all the most prescribed ICS are effective, but physicians should know devices and drugs doses in order to obtain an equivalence in terms of efficacy and safety issues when using each of the available molecules. Therefore, pharmacodynamics and pharmacokinetics of corticosteroids knowledge should be improved, and these aspects have been fully examined in the recently published Italian consensus document [10]. Our study has some limitations. First of all, even if university pediatricians are far less numerous than primary care and hospital pediatricians in our country, their response rate was particularly low, so that the subgroups division of the participants on the basis of work settings could not be completely representative of the national situation. Moreover, pediatric pulmonologists are almost exclusively university professionals in Italy, so that, since their responses may be missing, our results may underestimate good clinical practice in our country in terms of ICS prescription. Another limit is that we couldn’t check diagnostic criteria in our sample. However, we are confident that diagnoses were correct, since the diseases included in the questionnaire are extremely common in childhood, so that every pediatrician should be able to recognize and treat them, considering also a similar background training across the country. Moreover, regarding the geographical distribution of the responders, the number of responses from the various Regions reliably reflects the number of pediatricians and inhabitants from each Region. However, we recognize that in the smallest regions response rates are particularly low and this could represent another limitation to our study. Last but not least, our questionnaire was not validated and was very simple, in order to obtain higher response rates. Regarding drugs, it was not possible to include all the available treatments for the different diseases and we had to choose to narrow the field on the most used ones in childhood (nasal ICS and antihistamine in AR, ICS in asthma and laryngitis, ICS and antileukotrienes in preschool wheezing).

## Conclusions

To our knowledge, this is the first survey on pediatricians’ ICS prescription habits performed in Italy and it should be considered as a pilot study. Our study confirmed that AR is mainly treated following guidelines, while ICS are still too much used in the management of acute asthma, even at high doses. Unfortunately, high doses are commonly used also in the long-term management of these patients. Data emerging from our survey suggest that ICS prescription habits and good clinical practices in Italy should be improved, especially in the treatment of asthma. Moreover, further and more detailed surveys should be performed on this subject, to shed more light on differences in prescription habits on the basis of pediatricians’ work settings and geographical distribution.

## Supplementary Information

**Additional file 1:.** Italian pediatric scientific societies and professional associations involved in the consensus statement and web-based survey projects on ICS use in childhood.

**Additional file 2: **STROBE Statement—Checklist of items that should be included in reports of ***cross-sectional studies***

## Data Availability

Not applicable.

## Abbreviations

AR: Allergic Rhinitis
ICS: Inhaled CorticoSteroids
pMDI: Pressurized Metered Dose Inhaler
SIP: Società Italiana di Pediatria (Italian Society of Pediatrics)
