# Supplementary material for: Inhaled corticosteroids use in childhood respiratory diseases: an italian survey on pediatricians’ prescription habits
Source: Ital J Pediatr. 2021 Feb 15;47:34. doi: 10.1186/s13052-021-00988-8 (PMC7885514; doi:10.1186/s13052-021-00988-8)
Supplement: Supplementary file 1 — Additional file 1:. Italian pediatric scientific societies and professional associations involved in the consensus statement and web-based survey projects on ICS use in childhood. [file 13052_2021_988_MOESM1_ESM.docx]

**Additional File 1** – Italian pediatric scientific societies and professional associations involved in the consensus statement and web-based survey projects on ICS use in childhood.

- **FIMP (Federazione Italiana Medici Pediatri)**

Presidente: Paolo Biasci

Website: [https://www.fimp.pro](https://www.fimp.pro/)

- **SIAIP (Società Italiana di Allergologia ed Immunologia Pediatrica)**

Presidente: Gianluigi Marseglia

Website: [https://www.siaip.it](https://www.siaip.it/)

- **SICuPP (Società Italiana delle Cure Primarie Pediatriche)**

Presidente: Paolo Becherucci

Website: [https://www.sicupp.org](https://www.sicupp.org/)

- **SIMA (Società Italiana di Medicina dell’Adolescenza)**

Presidente: Gabriella Pozzobon

Website: [http://www.medicinadelladolescenza.com](http://www.medicinadelladolescenza.com/)

- **SIMEUP (Società Italiana di Medicina Emergenza Urgenza Pediatrica)**

Presidente: Riccardo Lubrano

Website: [https://www.simeup.it](https://www.simeup.it/)

- **SIMRI (Società Italiana per le Malattie Respiratorie Infantili)**

Presidente: Giorgio Piacentini

Website: [www.simri.it](http://www.simri.it)

- **SIP (Società Italiana di Pediatria)**

President: Alberto Villani

Website: [www.sip.it](http://www.sip.it)

- **SIPPS (Società Italiana di Pediatria Preventiva e Sociale)**

Presidente: Giuseppe Di Mauro

Website: [https://www.sipps.it](https://www.sipps.it/)
